# Diagnosed but Not Undiagnosed Diabetes Is Associated with Depression in Rural Areas

**DOI:** 10.3390/ijerph13111136

**Published:** 2016-11-14

**Authors:** Zhao Li, Xiaofan Guo, Hongkun Jiang, Guozhe Sun, Yingxian Sun, Maria Roselle Abraham

**Affiliations:** 1 Department of Cardiology, the First Hospital of China Medical University, Shenyang 110001, China; zhaolisy@outlook.com (Z.L.); guoxiaofanl1986@outlook.com (X.G.); melichian@aliyun.com (G.S.); 2 Department of Pediatrics, the First Hospital of China Medical University, Shenyang 110001, China; jianghongkun1975@sina.com; 3 Department of Cardiology, Johns Hopkins University, Baltimore, MD 21218, USA; meilichian3@gmail.com

**Keywords:** depression, diagnosed diabetes, population

## Abstract

*Background*: There is a lack of study on the relation between undiagnosed diabetes and depression in the general population. *Methods*: A total of 11,531 adults were examined using a multistage cluster sampling method to select a representative sample of individuals who were at least 35 years old. Subjects were classified into three groups: no diabetes (ND), diagnosed diabetes (DD), and undiagnosed diabetes (UD). The participants were surveyed with the Patient Health Questionnaire-9 (PHQ-9). *Results*: Of all the 11,531 participants, the prevalence of depression was higher in the DD group than in the other two groups. Multi variable logistic regression analyses show that the DD group had significantly higher odds for depression compared with the ND group (*p* < 0.01), while the UD group showed no significant differences compared to the ND group. Subgroup analyses show that diagnosed diabetes in subjects with a lower educational level, compared with subjects with an educational level of high school or above, had higher odds for a PHQ-9 score ≥5 (*p* < 0.01). *Conclusion*: In this general population, diagnosed but not undiagnosed diabetes was significantly associated with depression. Much higher odds for depression were found among diagnosed diabetic individuals with a lower level of education.

## 1. Introduction 

The association between diabetes and depression has been well explored [1,2,3], showing that there is a bidirectional relationship between depression and type 2 diabetes [4,5,6]. The latest studies report that the relationship between diabetes and depression depends on whether or not diabetes has been clinically identified [7]. Further, scientists have argued that meeting clinical criteria for diabetes was not sufficient to explain increased depressive symptoms, whereas “knowing that one is diabetic” was associated with depression in diabetic subjects [8]. Evidence for this argument has been limited, however, particularly with regard to the general population.

Depression is burdensome and common in low- and middle-income countries (LMICs) and has received more attention in recent years [9,10]. Studies have warned that depression among people with diabetes may increase as the incidence of diabetes shifts from affluent to lower-income groups [11]. One study has also reported a large increase in psychogenic and metabolic disease in rural China, where the prevalence of depressive symptoms associated with diabetes was high [12]. However, there was little awareness of the disease or its symptoms among this rural population, where few diabetic individuals had been identified or diagnosed. That is, in this area of China, there was a high incidence of undiagnosed diabetes. Until now, no report has focused on the relation between undiagnosed diabetes and depression in China’s rural population. We therefore designed our study to comprise a general Chinese population to evaluate the hypothesis that the diagnosis of diabetes is associated with depression and to examine related factors prevalent in rural areas.

## 2. Methods

### 2.1. Study Population

The methods used in this study have been previously published [13]. Briefly, from January 2012 to August 2013, a representative sample of individuals who were at least 35 years old was selected adopting a multistage, stratified, random cluster sampling scheme in rural areas in the province of Liaoning. Pregnancy and malignant tumors were used as exclusion criteria. The exclusions also included the presence of another major axis I psychiatric disorder such as bipolar disorder, schizophrenia, schizoaffective disorder, substance abuse and dementia, and other neurological disorders such as stroke, seizure disorder, Parkinson’s disease, and multiple sclerosis. All the eligible permanent residents from each village were invited to participate, which was a total of 14,016 invited participants. Of those, 11,956 participants agreed to participate, and the response rate was 85.3%. The study was approved by the Ethics Committee of China Medical University (Shenyang, China, AF-SDP-07-1,0-01). All procedures were performed in accordance with ethical standards. Written consent was obtained from all participants after they had been informed of the objectives, benefits, and medical factors. All participants signed a confidentiality agreement for the protection of their personal information. If the participants were illiterate, we obtained written informed consent from their proxies. We used baseline data in this report, and only participants who had complete data sets for the variables analyzed in the study were included, yielding a final sample size of 11,531 individuals (5334 males and 6197 females). 

### 2.2. Data Collection 

Using a standard questionnaire and a face-to-face interview, data were collected by cardiologists and trained nurses during a single clinic visit. Information about demographics, family history of diabetes, lifestyle risk factors, dietary habits, family income, history of chronic diseases, and any medication used over the past two weeks were obtained. Fasting plasma glucose (FPG), as well as other routine blood biochemical indexes, were analyzed enzymatically using an auto-analyzer (Olympus AU640 Auto-Analyzer; Olympus Corp., Kobe, Japan). All laboratory equipment was calibrated, and blinded duplicate samples were used. Detailed data collection and measurements methods of this study have been described elsewhere [14,15,16].

### 2.3. Definitions

Diabetes was defined as a fasting plasma glucose (FPG) value of ≥7.0 mmol/L or a previous diagnosis of diabetes by a medical practitioner [17]. 

Diagnosed diabetes: Investigators collected information by asking: “Have you been diagnosed with diabetes or high blood sugar by a doctor?” Respondents who answered yes to this question were classified as having diagnosed diabetes (DD).

Undiagnosed diabetes: Respondents who were identified as having diabetes (on the basis of a FPG value of ≥7.0 mmol/L) who answered no to the preceding question were classified as having undiagnosed diabetes (UD) [18].

No diabetes: Respondents who were identified as not having diabetes and who answered no to the preceding question were classified as having no diabetes (ND).

### 2.4. Measuring Depression Symptoms

In this study, we used PHQ-9, a questionnaire widely used in primary health centers, to assess depression symptoms [19,20]. A detailed application procedure for this questionnaire has been described elsewhere [20]. In general, PHQ-9 scores range from 0 to 27, with scores of ≥5, ≥10, and ≥15 representing mild, moderate, and severe levels of depression severity [21,22]. The psychometric properties of the PHQ-9 are well documented [23]. 

### 2.5. Statistical Analysis

Descriptive statistics were calculated for all variables, including continuous variables (expressed as mean values and standard deviations) and categorical variables (expressed as numbers and proportions). Differences among the categories were analyzed using non-parametric tests or the χ2 test appropriately. Multivariate logistic regression analyses were used to identify correlates of PHQ-9 scores ≥5 or PHQ-9 scores ≥10. We created 4 models for multivariate logistic regression analyses: Model 1 was adjusted for age, gender, and race group; Model 2 was adjusted for factors in Model 1 plus education level, family income, marital status, and family history of diabetes; Model 3 was adjusted for factors in Model 2 plus body mass index (BMI), diet score, sleep duration, current smoking, drinking status, and physical activity; Model 4 was a multivariable model further adjusted for factors in Model 3 plus history of chronic disease and medication. We chose Model 4 as the final regression model on which to base our further discussion and conclusions. The association strengths are expressed as odds ratios (ORs) and corresponding 95% confidence intervals (CIs). All the statistical analyses were performed using SPSS version 22.0, and *p* values < 0.05 were considered statistically significant. 

## 3. Results 

### 3.1. Basic Characteristics of the Study Population

Of the 11,531 participants, 46.1% were males; the mean age was 54 years and ranged from 35 to 93 years. According to the definition, of all the participants, 529 (4.6%) and 692 (6.0%) were in the DD and UD groups, respectively; 10,310 were in the ND group (89%). Table 1 shows the characteristics of the groups studied. No significant differences were found among the three groups in the variables of family income, history of renal disease, sleep duration, and physical activity. Age, gender, marital status, educational level, and history of heart disease or stroke pointed to significant differences among the three groups (all *p* < 0.05). The mean PHQ-9 scores of participants in the ND, DD, and UD groups were 2.9 ± 3.6, 4.6 ± 4.7, and 3.0 ± 3.7, respectively (*p* < 0.01).

### 3.2. Prevalence of Depression 

**Figure 1:**
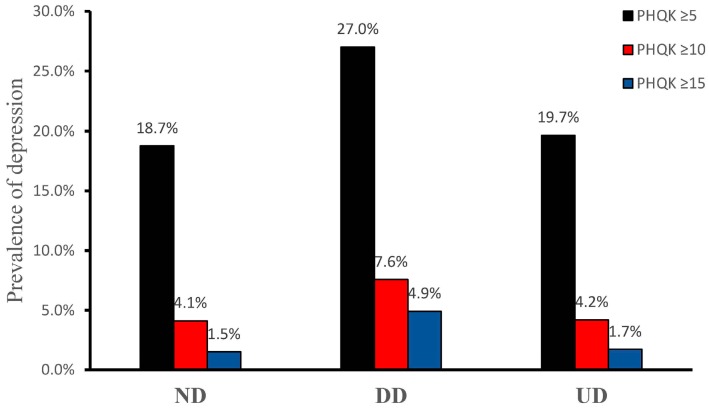
Prevalence of depression in different groups (*p* < 0.001). Abbreviations: ND: No diabetes; DD: Diagnosed diabetes; UD: Undiagnosed diabetes; PHQK: Patient health questionnaire score.

**Figure 2:**
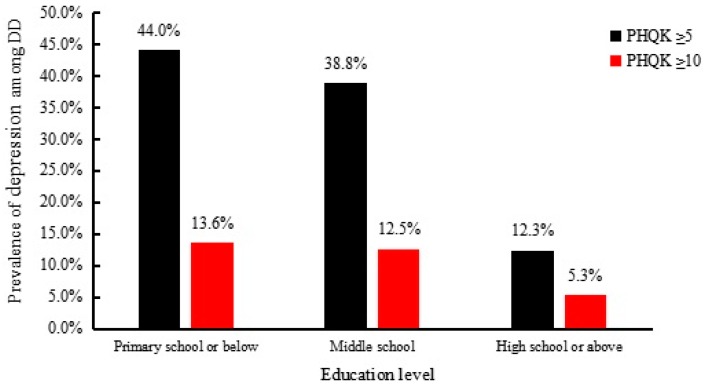
Prevalence of depression in educational level (*p* < 0.001). Abbreviations: PHQK: Patient health questionnaire score; DD: Diagnosed diabetes.

Among the ND group, the prevalence of mild, moderate, and severe depression was 18.7%, 4.1%, and 1.5%, respectively. The prevalence among the DD group for mild, moderate and severe depression was 27.0%, 7.6%, and 4.9%, respectively. For the UD group, the prevalence of mild, moderate, and severe depression was 19.7%, 4.2%, and 1.7%, respectively. The prevalence of depression was higher in the DD group than in the other two groups (Figure 1). Subgroup analysis shows that, among the DD population, the prevalence of depression was significantly different in terms of education level (*p* = 0.002). Participants who had not completed or gone any further than primary school had a higher prevalence of depression as compared with those in the two higher educational levels (Figure 2).

### 3.3. Results from Multivariate Logistic Regression Analyses 

Taking PHQ-9 scores of ≥5 or ≥10 as the dependent variables and the ND, DD, and UD groups as independent variables, multivariate logistic regression analyses were applied to assess the association between diabetes and depression. Model 1 was adjusted for age, gender, and racial group; Model 2 was adjusted for factors in Model 1 plus education level, family income, marital status and family history of diabetes; Model 3 was adjusted for factors in Model 2 plus BMI, diet score, sleep duration, current smoking, drinking status, and physical activity; Model 4 was a multivariable model further adjusted for factors in Model 3 plus history of chronic disease and medication. Table 2 shows that the DD group had significantly higher odds for depression compared with the ND group in the four models (all *p* < 0.01), while the UD group showed no significant difference compared with the ND group.

We did subgroup analyses, as shown in Table 3, using multiple regression among the DD population adjusted for age, race, gender, marital status, educational level, annual income, family history of diabetes, current smoking status, BMI, diet score, physical activity, plus sleep duration and history of chronic disease. Results show that, compared with subjects who had completed high school or more, people at lower educational levels have higher odds for PHQ-9 scores of ≥5 and ≥10.

## 4. Discussion

In the present study, we first evaluated depression among the DD and UD groups in a general population aged 35 or older from rural China. We found that, compared with the ND participants, undiagnosed diabetes was not associated with increased odds for depression. Diagnosed diabetes, on the other hand, pointed to significantly higher odds for depression, as compared with the ND participants. Furthermore, after multiple regression analyses, we found a lower amount of participants with diagnosed diabetes.

Studies have confirmed that depression is associated with a number of chronic diseases, including diabetes [24,25,26,27]. Previous reports indicate that people with diabetes have twice the risk of experiencing depression or serious psychological distress, as do those without diabetes [28]. However, in the present study, there was no significant difference in PHQ-9 scores between people without diabetes (ND) and people with diabetes (UD) who had not been diagnosed by a medical practitioner. Moreover, multiple regression analyses adjusted for confounding factors also showed that it was DD but not UD that posed a significantly higher risk for depression compared with ND. Our results are similar to those of Olvera et al. who reported that, in a study of Mexican Americans, the DD group had significantly higher scores on the self-report depression scale (CES-D) than either the ND or UD groups, whereas the ND and UD groups showed no significant difference [8]. Our results also agreed with those reported by Huaqing Liu, who examined the prevalence for depression among DD individuals as compared with a UD population [29]. In contrast, a study from Netherlands reported by Maaike Meurs reported a different result in that both DD and UD populations were significantly associated with higher odds for depression, as compared with a ND group [30]. Associations between diabetes and depression may be different among Asians as compared with Western populations [31]. Alternatively, as discussed by Maaike Meurs, factors indicating the severity of diabetic disease may have affected the prevalence of depression among DD versus UD patients [30]. However, these varying results suggest that the relationship between diabetes and depression is far more complicated than we had thought. We arrived at the conclusion that educational level affected the prevalence of depression; we also noticed that the subjects with higher family incomes were somewhat less likely to be depressed. These factors may differ from those clinical factors that can be controlled by doctors. Our study reminds us that much more attention from both society and the government should be given to the effects of socioeconomic factors. However, much further study is needed to elucidate this conclusion.

Our further analyses revealed that, in rural areas, a lower educational level was associated with depression in the DD group. This is consistent with the recent prospective observational study demonstrating that a lower education level at baseline was associated with significantly higher depression scores among individuals with diabetes [32]. Thus, combining all of our earlier results, we recommend that subjects newly diagnosed with diabetes should be screened for depression and that specific interventions for depression should be provided, especially for those with less education.

There are several limitations in our research. First, we did not confirm a definitive diagnosis of depression. Second, we did not perform detailed quantitative analyses of the factors indicating diabetes disease severity, although this is an important topic for future research. In the subgroup of the DD population, we used cutoff PHQ-9 scores of ≥5 and ≥10 for our multiple regression analyses. PHQ-9 scores ≥15 in this subgroup were too low, and we were unable to obtain the corresponding data. The results might have been different. Finally, even though we informed subjects with undiagnosed diabetes immediately of their condition and provided strong recommendations to see a specialist, a follow-up study in this regard is yet to be performed.

## 5. Conclusions

Based on this general population from China, we found that diagnosed but not undiagnosed diabetes was significantly associated with depression. Much higher odds for depression were found among diagnosed diabetic individuals with a lower level of education. In the future, such individuals should be given more attention and care.

## Figures and Tables

**Table 1:** Baseline characteristics of study population (*n* = 11531).

Variables	ND (*n* = 10,310)	DD (*n* = 529)	UD (*n* = 692)	*p*-Value
Age (year)	53.4 ± 10.6	58.4 ± 9.0	57.0 ± 10.1	<0.01
Male gender	4799 (46.5)	186 (35.2)	349 (50.4)	<0.01
Female gender	5511 (53.5)	343 (64.8)	343 (49.6)	
Marital status				
Married	9465 (91.8)	479 (90.5)	618 (89.3)	0.01
Single	185 (1.8)	5 (0.9)	11 (1.6)	
Widowed	660 (6.4)	45 (8.5)	63 (9.1)	
Race of Han	9764 (94.7)	505 (95.5)	649 (93.8)	0.42
others	546 (5.3)	24 (4.5)	43 (6.2)	
Education				<0.01
primary school or below	5021 (48.7)	322 (60.9)	400 (57.8)	
middle school	4297 (41.7)	153 (28.9)	244 (35.3)	
high school or above	992 (9.6)	54 (10.2)	48 (6.9)	
Family income (CNY/year)				
≤5000	1258 (12.2)	74 (14.0)	98 (14.2)	0.39
5000–20,000	5625 (54.6)	285 (53.9)	378 (54.6)	
>20,000	3427 (33.2)	170 (32.1)	216 (31.2)	
Smoking	3687 (35.8)	108 (20.4)	264 (38.2)	<0.01
Drinking	2328 (22.6)	63 (11.9)	197 (28.5)	<0.01
Physical activity				1.00
Low	3076 (29.8)	156 (29.5)	205 (29.6)	
Moderate	6653 (64.5)	343 (64.8)	449 (64.9)	
High	581 (5.6)	30 (5.7)	38 (5.5)	
BMI (kg/m^2^)	24.6 ± 3.6	26.2 ± 3.6	26.2 ± 3.8	<0.01
Sleep duration (h/d)	7.3 ± 1.9	7.1 ± 2.1	7.3 ± 2.7	0.09
Diet score	2.3 ± 1.1	2.0 ± 1.1	2.3 ± 1.1	<0.01
History of hypertension				
no	8269 (80.2)	258 (48.8)	445 (64.3)	0.39
yes	2041 (19.8)	271 (51.2)	247 (35.7)	
History of heart disease ^a^				
no	9374 (90.9)	509 (96.2)	641 (92.6)	<0.01
yes	936 (9.1)	20 (3.8)	51 (7.4)	
History of stroke				
no	9724 (94.3)	514 (97.2)	659 (95.2)	0.01
yes	586 (5.7)	15 (2.8)	33 (4.8)	
History of renal disease				
no	7730 (75.0)	380 (71.8)	522 (75.4)	0.25
yes	2580 (25.0)	149 (28.2)	170 (24.6)	
Medication used ^b^				
no	6778 (65.7)	429 (81.1)	521 (75.3)	<0.01
yes	3532 (34.3)	100 (18.9)	171 (24.7)	
PHQ-9 score	2.9 ± 3.6	4.6 ± 4.7	3.0 ± 3.7	<0.01

Data are expressed as the mean ± SD or as *n* (%). Abbreviations: ND: No diabetes; DD: Diagnosed diabetes; UD: Undiagnosed diabetes; CNY: China Yuan (1 CNY = 0.157 USD); BMI: Body mass index. **^a^** Including coronary heart disease, arrhythmia, and heart failure; **^b^** Indicating any self-reported medication used in the past two weeks.

**Table 2:** Multivariable logistic regression analyses for depression among the total population.

Model	Variables	PHQK ≥ 5			PHQK ≥ 10		
		OR	95% CI	*p*-Value	OR	95% CI	*p*-Value
Model 1	ND	1.00 (reference)			1.00 (reference)		
	DD	1.70	1.412.04	<0.01	1.89	1.43–2.49	<0.01
	UD	1.01	0.84–1.21	0.95	0.96	0.69–1.34	0.81
Model 2	ND	1.00 (reference)			1.00 (reference)		
	DD	1.68	1.38–2.03	<0.01	1.93	1.45–2.57	<0.01
	UD	1.00	0.83–1.20	0.97	0.96	0.69–1.34	0.80
Model 3	ND	1.00 (reference)			1.00 (reference)		
	DD	1.75	1.43–2.12	<0.01	1.94	1.44–2.61	<0.01
	UD	1.06	0.88–1.27	0.56	0.99	0.71–1.40	0.96
Model 4	ND	1.00 (reference)			1.00 (reference)		
	DD	1.62	1.32–1.98	<0.01	1.70	1.25–2.31	0.00
	UD	1.04	0.86–1.25	0.71	0.92	0.65–1.31	0.66

Model 1: Adjusted for age, sex, and race; Model 2: Adjusted for factors in Model 1, education level, family income, marital status, and family history of diabetes; Model 3: Adjusted for factors in Model 2, body mass index, diet score, sleep duration, current smoking, drinking status, and physical activity; Model 4: Adjusted for factors in Model 3, history of chronic disease, and any medication. Abbreviations: PHQK: Patient health questionnaire score; DD: Diagnosed diabetes; UD: Undiagnosed diabetes; ND: No diabetes. OR: Odds ratio; 95% CI: 95% Confidence interval.

**Table 3:** Multivariable logistic regression analyses for depression among diagnosed diabetes population.

Variable	PHQ ≥ 5			PHQ ≥ 10		
	*p*-Value	OR	95% CI	*p*-Value	OR	95% CI
Age (year)	0.86	1.00	0.98–1.03	0.29	1.02	0.98–1.06
Male gender	0.37	1.27	0.76–2.12	0.13	1.87	0.83–4.25
Race of Han	0.72	1.19	0.47–3.02	0.72	1.27	0.34–4.82
Marital status						
Married	0.40		1.00 (reference)	0.97		1.00 (reference)
Single	0.54	0.80	0.40–1.62	0.81	1.12	0.43–2.92
Widowed	0.19	0.20	0.02–2.16	1.00	0.00	0
Family income (CNY/year)						
≤5000	0.22		1.00 (reference)	0.33		1.00 (reference)
5000–20,000	0.31	0.75	0.42–1.31	0.50	0.78	0.37–1.62
>20,000	0.09	0.58	0.31–1.09	0.15	0.53	0.22–1.26
Education						
High school or above	0.01		1.00 (reference)	0.60		1.00 (reference)
Primary school or below	0.00	3.94	1.65–9.45	0.48	1.58	0.44–5.71
Middle school	0.00	3.93	1.62–9.56	0.33	1.91	0.52–7.10
BMI (kg/m^2^)	0.24	0.97	0.91–1.02	0.53	0.97	0.90–1.06
Diet score	0.01	0.80	0.67–0.95	0.03	0.75	0.58–0.97
Family history of diabetes	0.55	0.89	0.59–1.32	0.48	0.80	0.44–1.47
Smoking	0.03	1.75	1.05–2.94	0.26	1.55	0.72–3.36
Drinking	0.98	0.99	0.50–1.96	0.83	1.14	0.37–3.54
Totalsleep	0.00	0.85	0.76–0.94	0.07	0.87	0.76–1.01
Physical activity						
Low	0.77		1.00 (reference)	0.46		1.00 (reference)
Moderate	0.50	0.86	0.57–1.32	0.84	1.07	0.58–1.97
High	0.98	1.01	0.43–2.40	0.26	0.40	0.08–1.97
History of heart disease ^a^	0.77	1.08	0.65–1.79	0.69	1.16	0.56–2.38
History of stroke	0.48	0.77	0.37–1.60	0.34	0.54	0.15–1.92
Medication used ^b^	0.36	0.77	0.45–1.34	0.14	0.50	0.20–1.27
History of hypertension	0.71	1.08	0.72–1.62	0.78	1.09	0.60–1.98
History of renal disease	0.42	1.19	0.78–1.83	0.87	1.05	0.57–1.93

Abbreviations: PHQ: Patient health questionnaire score; CNY: China Yuan (1 CNY = 0.157 USD); BMI: Body mass index. **^a^** Including coronary heart disease, arrhythmia, and heart failure; **^b^** Indicating any self-reported medication used in the past two weeks.
